# Biological function identification of phage holin Hol-4086 and treatment of *Staphylococcus aureus* infection

**DOI:** 10.3389/fmicb.2025.1499566

**Published:** 2025-02-25

**Authors:** Chenyu Zheng, Yaxin Di, Yingchun Wang, Huyang Zhao, Ruichong Wang, Guiwei Li, Xiaona Wang, Zhifu San, Yanping Jiang, Wen Cui, Jiaxuan Li, Li Wang, Xinyuan Qiao

**Affiliations:** ^1^Heilongjiang Key Laboratory for Animal Disease Control and Pharmaceutical Development, Department of Preventive Veterinary Medicine, College of Veterinary Medicine, Northeast Agricultural University, Harbin, China; ^2^Department for Radiological Protection, Heilongjiang Province Center for Disease Control and Prevention, Harbin, China; ^3^Institute of Rural Revitalization Science and Technology, Heilongjiang Academy of Agricultural Sciences, Harbin, China

**Keywords:** *Staphylococcus aureus*, phage, holin, phage perforin, phage therapy

## Abstract

The treatment of infections caused by drug-resistant *Staphylococcus aureus* has become increasingly difficult. In this study, the complete genome of phage 4086-1 against *S. aureus* was sequenced and shown to be 17,960 bp in size, with a GC content of 29.14%. Phylogenetic analysis demonstrated that phage 4086-1 exhibited a close relationship with the *Staphylococcus* phages SLPW, JPL-50, and LSA2366. BLAST analysis indicated that ORF12 of phage 4086-1 (termed Hol-4086) shares high identity with other reported phage-associated holins. Hol-4086 consists of 140 amino acids and exhibits high sequence identity with some members of the phage_holin_4_1 superfamily (18–125 amino acids). Hol-4086 was then expressed in *Escherichia coli* and detected using sodium dodecyl sulfate-polyacrylamide gel electrophoresis and western blotting. The results of a spot test demonstrated that Hol-4086 had substantial bacteriostatic effects on the host bacteria. *S. aureus* cells were exposed to Hol-4086 and observed using transmission electron microscopy and scanning electron microscopy. Bacterial cells treated with Hol-4086 showed ultrastructural and morphological changes. The detection of biofilm activity showed that Hol-4086 effectively inhibited and removed *S. aureus* biofilms. *In vivo*, treatment with Hol-4086 significantly reduced the number of bacteria, relieved inflammatory responses, and alleviated pathological changes in the organs of infected mice 48 h after treatment. These results demonstrate that Hol-4086 exhibits promising antibacterial potential as an alternative therapy for the treatment of infections caused by *S. aureus*.

## Introduction

*Staphylococcus aureus* is a pathogen with remarkable adaptive power that causes diverse severe infections, such as pneumonia, bacteremia, scalded skin syndrome, toxic shock syndrome, endocarditis, and sepsis (Cheung et al., 2021; Cong et al., 2020; Nwabuife et al., 2021). The treatment of infectious diseases caused by pathogenic bacteria is largely based on the use of antibiotics. The introduction of antibiotics into clinical medicine in the mid-20th century significantly reduced morbidity and mortality from bacterial infections; while pathogenic bacteria did not disappear, they did become more manageable (Loganathan et al., 2021). However, the emphasis on this treatment has led to an overreliance on antibiotics, leading to the emergence of antibiotic resistance. Treatment of infections caused by *S. aureus* has become more difficult because of the emergence of drug-resistant strains. These include methicillin-resistant *S. aureus* and vancomycin-resistant *S. aureus* (Cong et al., 2020; Lakhundi and Zhang, 2018; Turner et al., 2019). There is an urgent clinical need for non-antibiotic immune-based approaches to treat these infections because increasing antibiotic resistance is a serious threat to public health.

Phages are viruses that infect bacteria and were first discovered by Frederik Twort in 1915 and Felix d’Herelle in 1917 (Salmond and Fineran, 2015; Jariah and Hakim, 2019; Aranaga et al., 2022; Hatoum-Aslan, 2021). Phages are the most abundant biological entities and are estimated to amount to a total number of 10^31^ particles (Hesse and Adhya, 2019; Wei et al., 2020). Phages play a vital role in the treatment of antibiotic-resistant infections, one of the greatest threats to global health. Phage therapy has some strengths over traditional antibiotic therapy, including host specificity, and it does not affect other commensals (Anyaegbunam et al., 2022; Kakasis and Panitsa, 2019). Despite the great potential of phages, several constraints limit their broad application and acceptance in clinical practice. The emergence of phage-insensitive bacterial mutants, the stability of various types of phages, and the quality and safety requirements of phage preparations must be addressed in the coming years of phage research to advance the development of phage therapy (Pires et al., 2020; Bhargava et al., 2021). Thus, phage-encoded proteins with antibacterial properties have attracted considerable interest. Phage-encoded lysis proteins (endolysins and holins) can cause the breakdown of the bacterial membrane. Phages with single-stranded genomes encode cleavage effectors that inhibit the biosynthesis of bacterial peptidoglycan. In contrast, release of the phage progeny of double-stranded DNA (dsDNA) phages is mediated by two proteins (holin and endolysin) responsible for cell envelope disruption. Once the lytic life cycle is complete and the virion particles mature inside the bacterial cell, holin forms pores in the inner membrane, allowing endolysin to enter the cell wall. Subsequently, peptidoglycan is degraded by endolysin molecules, resulting in cell death and release of phage particles (Gutiérrez et al., 2018; Moghadam et al., 2020). However, some holins not only participate in phage-mediated cell lysis, but also participate in the process of phage DNA injection into host bacteria, destroying the cell wall and membrane of host bacteria from outside, and have strong lytic activity against bacteria (Qi et al., 2022; Nobrega et al., 2018).

Recently, phage proteins such as endolysins and holins have been explored as potential antimicrobial agents. Phage endolysin LysGH15 effectively protects against *Staphylococcus epidermidis* infection in mice. The number of bacteria decreased significantly in the organs of mice, and it also improved the pathological changes in the organs (Zhang et al., 2018). Phage endolysin LysSAP26, a gene potentially encoding the lysin of phage SAP-26, exhibits extended antibacterial activity. In animal experiments, mice infected with *Acinetobacter baumannii* were protected by LysSAP26, resulting in a survival rate of 40% (Kim et al., 2020). In the mouse infection model, Ply5218 repeated dosing (6, 24, and 48 h post-infection) not only alleviated the clinical symptoms caused by *Streptococcus* in piglets, but also significantly reduced the bacterial loads and the level of interleukin-6 (IL-6) (Wang et al., 2019). Moreover, research on the phage Lysin CF-301 has demonstrated that it can effectively remove biofilms and kill bacteria (Schuch et al., 2017). Meanwhile, many reports have indicated that holin proteins can cause cells to lose viability by forming holes in the cell membrane, which is also lethal to bacteria *in vitro* (Farkašovská et al., 2004). It was confirmed that the HolGH15 protein has an extracellular antibacterial function that can inhibit *S. aureus* and *Pseudomonas aeruginosa*. The combined application of LysGH15 enhanced antibacterial ability (Song et al., 2016).

In the present study, we isolated *S. aureus* phage 4086-1 from pig fecal samples (Tang, 2019) and analyzed its complete genome sequence. A putative holin protein from phage 4086-1 (named Hol-4086) was isolated and expressed in *E. coli*. The antibacterial effects of Hol-4086 were evaluated in vitro and *in vivo*, providing evidence for the feasibility of phage therapy for *S. aureus* infections.

## Materials and methods

### Bacterial strains and phage

*Staphylococcus aureus* CVCC 546 was purchased from the China Veterinary Culture Collection Center. *S. aureus* ATCC 43300 was purchased from the American Type Culture Collection Center. Phage 4086–1 was isolated from pig fecal samples and maintained in our laboratory. The phage 4086–1 was species-specific, attacking only *S. aureus*. *E. coli* pET-28a(+) and *E. coli* BL21 (DE3) were stored in our laboratory.

All animal experiments involved in this work were approved by the Animal Ethics Committee of Northeast Agricultural University, Harbin, China.

### Extraction and purification of phage DNA

The purified lysate was added to the culture of exponentially grown propagation host strain (*S. aureus* CVCC 546), and the mixture was incubated at 37°C in LB broth until the liquid was clear. All the phage cultures were collected for DNA extraction. Chloroform was added to the phage cultures at a final concentration of 0.5% and the cultures were incubated at 37°C for 1 h to allow complete dissolution. The obtained lysate was centrifuged at 10,000 r/min for 10 min, and the supernatant was collected and filtered. The filtrate was treated with DNase I (final concentration 50 U/mL, Sigma, St. Louis, MO, United States) and RNase A (final concentration 50 U/mL, Sigma, St. Louis, MO, United States) for 1 h at 37°C. Crude phage particles were treated with 1 mol/L NaCl in an ice bath for 1 h and then concentrated using 10% (w/v) polyethylene glycol 8,000. After centrifugation at 10000 r/min for 15 min, the samples were collected and resuspended in 1 mL SM buffer (0.05 M Tris–HCl, pH 7.5, 0.1 M NaCl, 0.017 M MgSO_4_, 0.01% gelatin). Phage DNA was obtained using phenol-chloroform extraction as follows (Sambrook et al., 1989). Add DNase I and RNase A to the crude phage sample to a final concentration of 10 μg/mL and 5 μg/mL respectively, and incubate at 37°C for 1 h. EDTA (pH 8.0) was then added to a final concentration of 20 mM. Further, incorporate proteinase K and SDS to final concentrations of 50 μg/mL and 10% respectively, and incubate at 56°C for 1 h. Sequentially, add equal volumes of Tris-saturated phenol, phenol-chloroform-isoamyl alcohol (25:24:1), and chloroform. The mixture was stirred gently for 30 s, centrifuged at 5000 r/min for 5 min, and the supernatant was collected. Subsequently, add an equal volume of ice-cold ethanol and 1/10 volume of 3 M NaAc, and keep at −20°C overnight. Centrifuge at 12,000 r/min for 20 min at 4°C. Wash the precipitate with pre-cooled ethanol. Centrifuge and discard the supernatant. After drying at room temperature, dissolve in sterile ultrapure water and store at −20°C.

### Sequencing and analysis of phage genome

Purified phage genomic DNA was prepared for next-generation sequencing to obtain the phage 4086-1 sequence. The putative function of each open reading frame (ORF) was predicted and annotated using BLAST software. The amino acid sequences of DNA polymerase were used to construct neighbor-joining phylogenetic trees using MEGA X 1.0.2.0.

### Bioinformatics analysis of Hol-4086

The DNA and protein sequences of the holin protein (Hol-4086) in phage 4086-1 were analyzed using BLAST. The TMHMM 2.0 Server^1^ was used to predict the transmembrane helices. Sequence alignment and Hol-4086 analysis were performed using BLAST^2^. The conserved domain was predicted using the NCBI CDD (Conserved Domains Database) search tool^3^.

### Expression of Hol-4086

The primers holF (5′-CGCGGATCCATGAATGAGGTAAAATTAAGATTTACG-3′) and holR (5′-CGAGCTCTTATCTATCTTCTCTTGGTCGTTCA-3′) were designed based on the complete sequence of the Hol-4086 gene. The reaction comprised of a 5 min denaturation at 94°C followed by 30 cycles of 30 s at 94°C, 30 s at 61°C, and 30 s at 72°C, and a final extension for 10 min at 72°C. The amplification products were inserted into the BamH I and Sac I sites of the pET-28a(+) vector. The recombinant plasmid, pET-Hol-4086, was transformed into competent *E. coli* BL21 (DE3) cells. A single positive colony was cultured in LB broth with 100 μg/mL ampicillin and grown to early mid-log phase (OD_600_ 0.4–0.6). Next, the mixture was induced with 1 mM isopropyl *β*-d-1-thiogalactopyranoside (IPTG) at 30°C for 8 h at 180 rpm. The precipitate was washed three times with phosphate buffered saline (PBS) (pH 7.4) and disrupted by sonication (4 s pulse, 4 s rest over 10 min) in an ice-cold water bath before centrifugation at 12,000 r/min. The supernatant was filtered using a 0.22 μm filter. All samples were stored for sodium dodecyl sulfate-polyacrylamide gel electrophoresis (SDS-PAGE) and western blotting analysis.

### Lytic activity of Hol-4086

The lytic activity of Hol-4086 was detected using spot testing. The culture of *S. aureus* (CVCC 546) was centrifuged to discard the supernatant. 2.5% glutaraldehyde fixative was added to fix at 4°C for more than 2 h. The cells were washed 3 times with 0.1 M PBS for 10 min each time, and the supernatant was discarded. After gradient dehydration with ethanol for 10 min and replacement with tert-butyl alcohol, the samples were freeze-dried for 4 h. Morphological and ultrastructural changes in *S. aureus* cells were visualized after exposure to Hol-4086 by transmission electron microscopy (TEM) (H-7650; Hitachi) and scanning electron microscopy (SEM) (SU8010; Hitachi).

### Activity of Hol-4086 against *S. aureus* biofilm

To assess the efficacy of the holin in preventing adhesion of *S. aureus* to an abiotic surface, 50 μL *S. aureus* (mid-log phase, OD = 0.6) and 150 μL LB medium were added to the wells of a 96-well tissue culture plate. Wells containing only the LB medium were used as negative controls. Meanwhile, 100 μL PBS, antibiotics, or Hol-4086 lysate were added to the wells, respectively. After incubation at 37°C for 1, 4, 8, 12, 16, 20, and 24 h, the biofilms were stained with crystal violet. Finally, absorbance was recorded at 570 nm using a microplate reader.

To test the efficacy of the holin on removing *S. aureus* biofilm, after incubation at 37°C for 48 h to form a mature biofilm, the medium was discarded and the plate wells washed with PBS. Next, 200 μL PBS, antibiotics (ceftiofur sodium), or Hol-4086 lysate were added to the wells. After 1, 4, 8, 12, 16, 20, and 24 h incubation at 37°C, the microplate was stained with crystal violet, and the results were recorded as stated previously.

### Endotoxin test of Hol-4086

Ten healthy mice were randomly divided into two groups. The experimental group was injected with 250 μL lysate of Hol-4086. The mental state and death of the mice were observed and compared with the control group injected with the same dose of PBS to analyze whether lysate of Hol-4086 was toxic to mice.

### Activity of Hol-4086 against *S. aureus* infection *in vivo*

To assess the effect of Hol-4086 treatment on septicemia caused by *S. aureus* infection, BALB/c mice aged 6–8 weeks were injected intraperitoneally with Hol-4086 lysate, ceftiofur, or PBS buffer 1 h after injection with 2× minimum lethal dose (2 × 10^8^ CFU/mouse) of *S. aureus* bacterial suspension (*n* = 10 in each group). Mice uninfected or treated served as controls. Bacterial loads in the blood and vital organs were measured 48 h after treatment using serial dilution and plating techniques. The blood, hearts, lungs, livers, spleens and kidneys (100 mg) were selected and homogenized in 1 mL of PBS with TissueLyser II (QIAGEN, Hilden, Germany). The homogenate was serially diluted (1:10) with PBS and coated on the plate at 37°C for 12 h incubation, bacterial colonies (CFUs) were then counted.

The cytokines, including interferon alpha (IFN-α) and IL-6, were quantified using enzyme-linked immunosorbent assay (ELISA). Histopathological analyses were performed on the tissues of the main organs. The mice were euthanized 48 h after treatment, and their hearts, lungs, livers, spleens, and kidneys were removed and immediately placed in 4% formalin. The fixed tissues were stained with hematoxylin and eosin using conventional staining methods, and the samples were histologically examined under an optical microscope.

### Statistical analysis

SPSS version 20.0 (SPSS Inc., Chicago, IL, United States) was used for all statistical analyses. Two-way analysis of variance was used to compare the quantities of bacteria and levels of cytokines in blood and organ samples. Error bars represent standard deviation.

## Results

### General features of the phage 4086-1 genome

**Figure 1:**
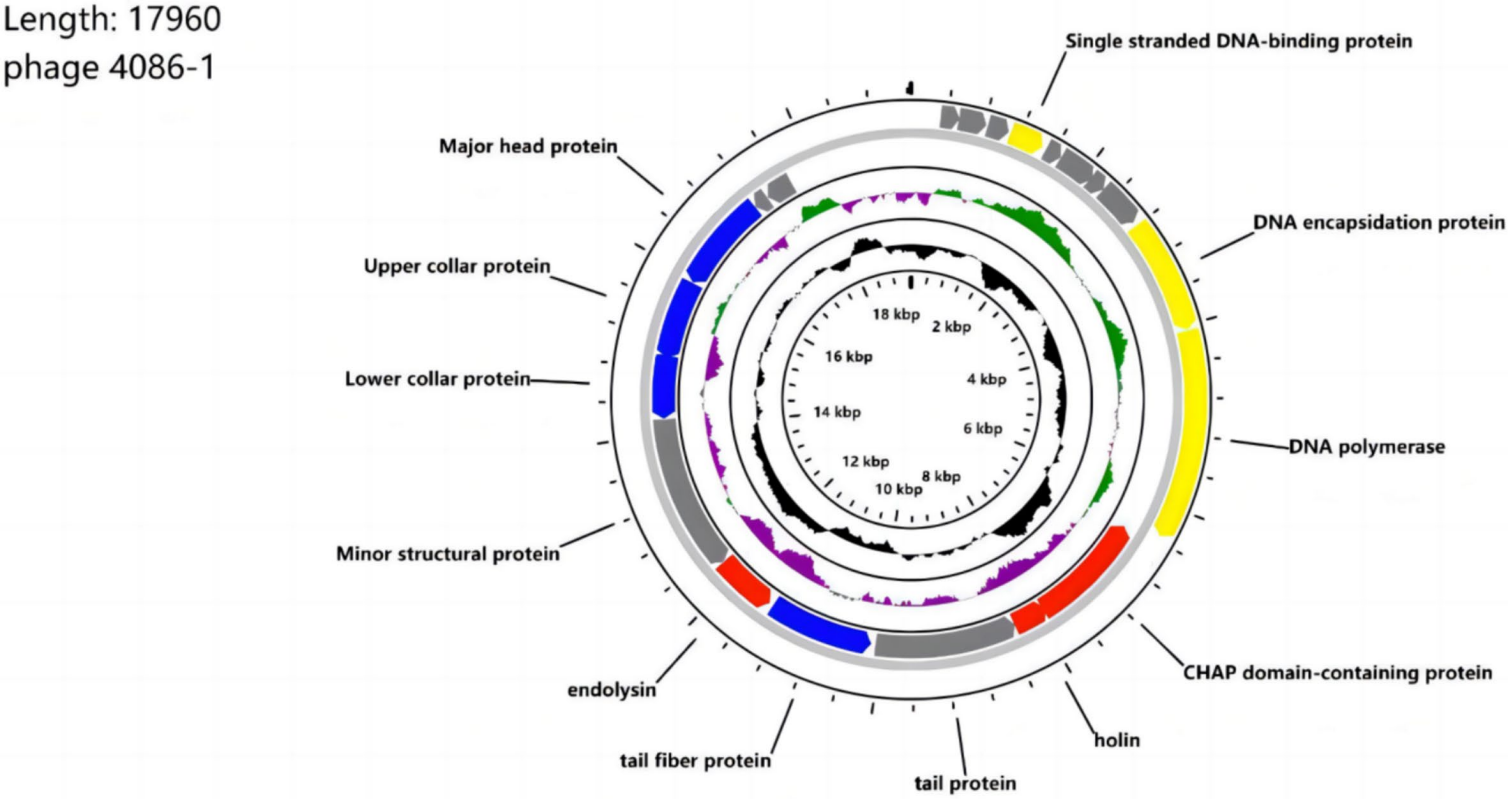
Genome map of phage 4086-1. The open reading frames (ORFs) are indicated by specific colors according to their functional categories. Consensus identities: red, lysis-related proteins; yellow, DNA and packaging-related proteins; blue; structural proteins; gray; hypothetical proteins. The GC skew is shown as inner circle holograms in green and purple. GC content is indicated by a black circular hologram.

The entire genome of phage 4086-1 was sequenced and annotated (Figure 1) (Accession number: PP541615). The phage genome consisted of circular double-stranded DNA with a genome size of 17,960 bp and a GC content of 29.14%. A total of 21 ORFs were predicted in the genome. Twelve ORFs were determined to have known functions and nine ORFs were classified as hypothetical proteins. ORF functions were predicted and classified into 4 categories: nucleotide metabolism-related, structure-related and packaging-related, lysis-related genes and hypothetical proteins genes. The results showed that ORF 12 probably encodes a holin protein (designated Hol-4086). Based on available databases, no functionally predicted genes related to virulence or lysogenicity were identified.

### Phylogenetic analysis of phage 4086-1

**Figure 2:**
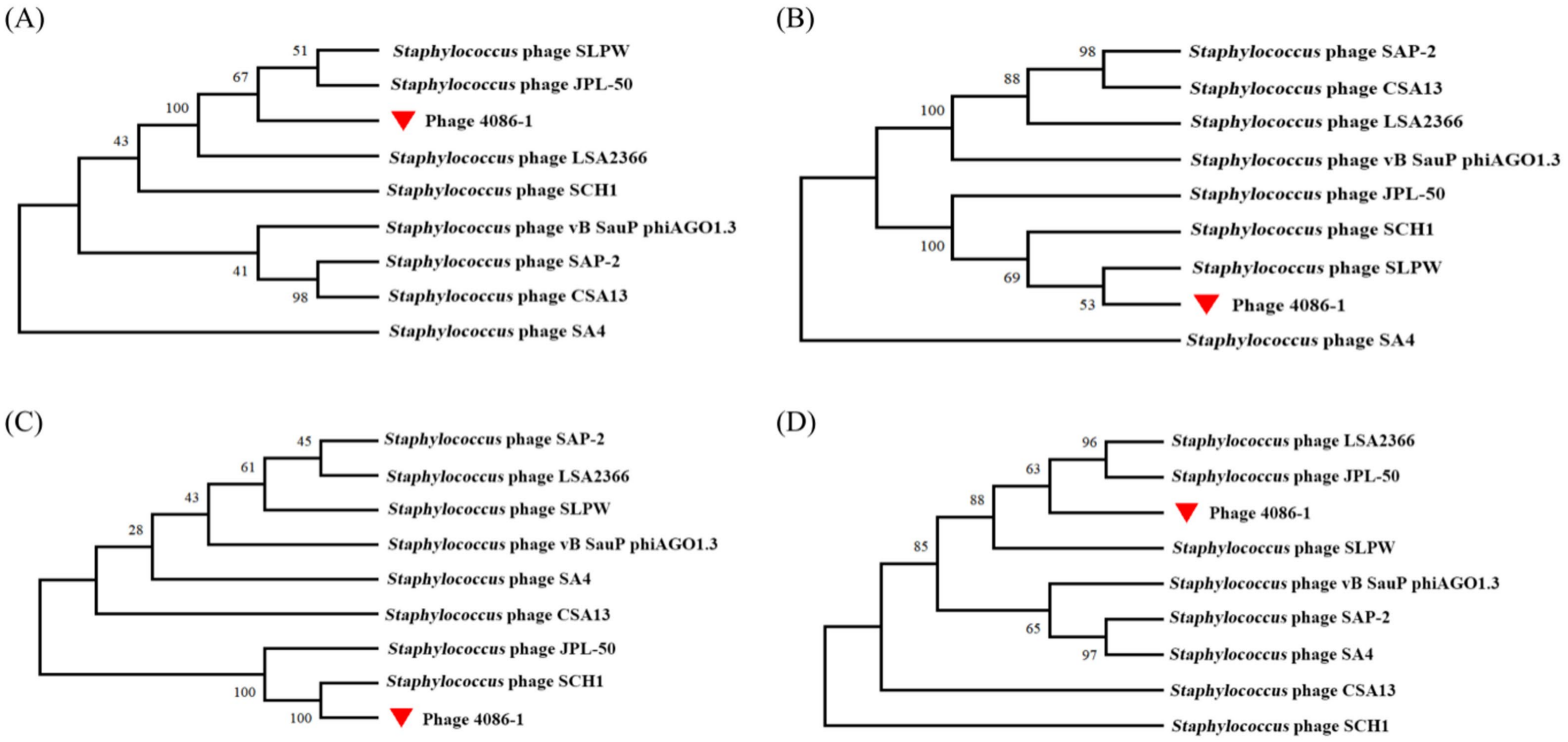
Phylogenetic analysis of the large terminase subunits from phage 4086-1 and other homologous phages. The phylogenetic analysis was constructed by comparing the sequence of the DNA polymerase **(A)**, major head protein **(B)**, tail fiber protein **(C)** and endolysin **(D)** to the corresponding sequences in homologous phages. The phage 4086-1 in the present study is highlighted using a red triangle.

Phylogenetic analysis demonstrated the genomic relationship of phage 4086-1 with other homologous phages. As shown in Figure 2, according to the amino acid sequence of the DNA polymerase (ORF10), phage 4086-1 is closely related to the *Staphylococcus* phages SLPW and JPL-50 (Figure 2A). Further analysis conducted based on the amino acid sequences of its major head protein (ORF 19), tail fiber protein (ORF 14) and endolysin (ORF 15). It was found that that the head protein of phage 4086-1 was closely related to *Staphylococcus* phages SLPW (Figure 2B). The tail protein of phage 4086-1 was closely related to *Staphylococcus* phages SCH1 (Figure 2C). And the endolysin of phage 4086-1 was closely related to *Staphylococcus* phages JPL-50 and LSA2366 (Figure 2D).

### Sequence analysis of Hol-4086

**Figure 3:**
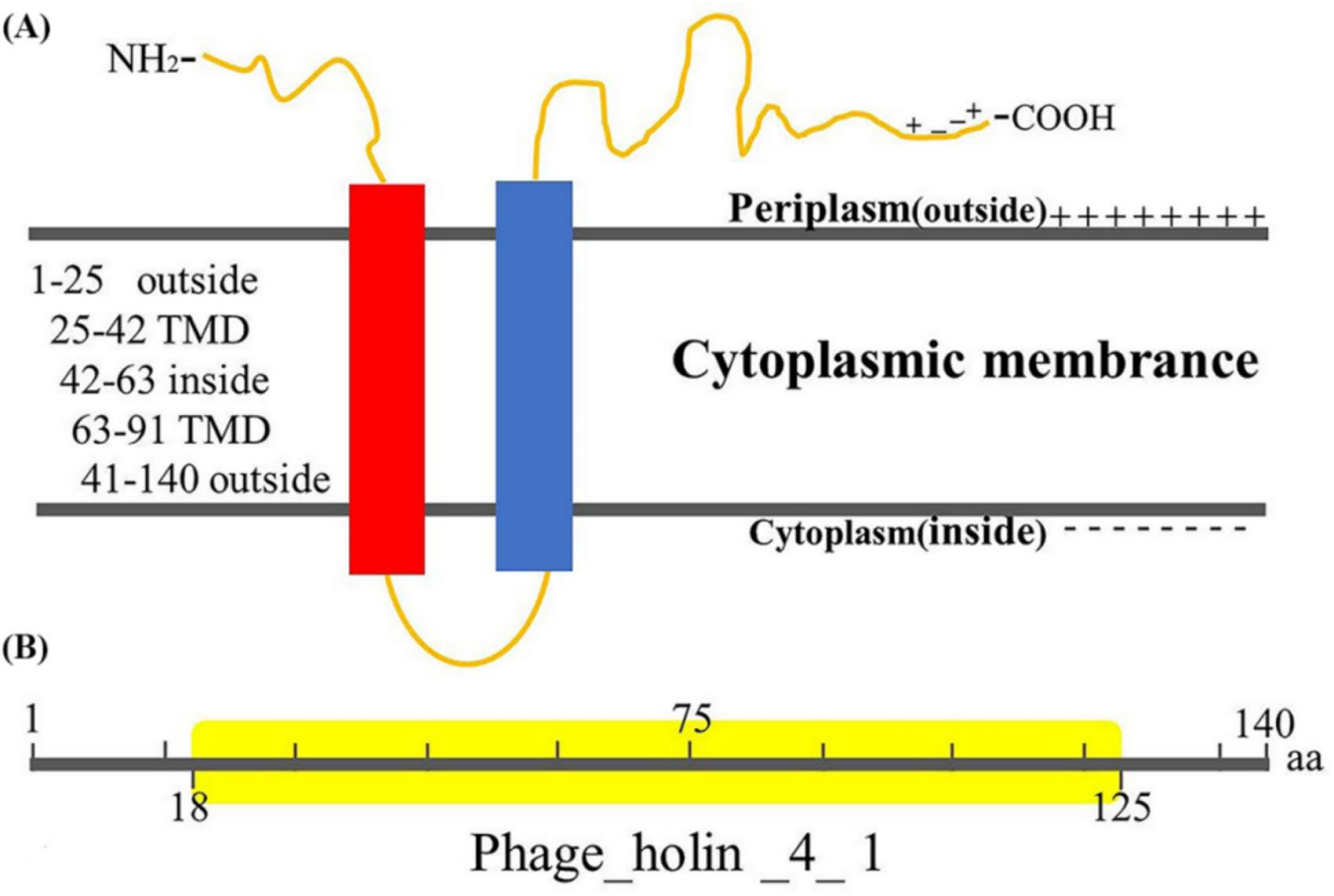
Predicted Hol-4086 protein topology. **(A)** Topological model of Hol-4086. Red box, TMD1; blue box, TMD2. **(B)** Putative conserved domain model of Hol-4086. Yellow; conserved domain. TMD, transmembrane domain.

The DNA sequence corresponding to or encoding Hol-4086 was compared to that of other published holins from *Staphylococcus* phages (Table 1). The results demonstrated that the holin region of phage 4086-1 (ORF 12) shared high sequence identity with different *Staphylococcus* phages (Accession number: PP477054). The results of TMHMM analysis suggested that Hol-4086 is a membrane protein with typical holin traits. This prediction indicated that Hol-4086 has two putative hydrophobic transmembrane domains (TMDs) with cytoplasmic N- and C-termini (Figure 3A). As shown in Figure 3B, Hol-4086 consists of 140 amino acids and exhibits high sequence identity with some members of the phage_holin_4_1 superfamily (18–125 amino acids).

**Table 1:** Homology analysis of Hol-4086.

Protein	Accession number	Source	Identity (%)
Holin	YP_009004302.1	*Staphylococcus* phage GRCS	100.00
Putative holin	YP_004957427.1	*Staphylococcus* phage S24-1	99.29
Holin	YP_009816547.1	*Staphylococcus* phage Pabna	99.29
Holin	QFG06614.1	*Staphylococcus* phage Portland	99.29
Putative holin	BAL42320.1	*Staphylococcus* phage S13’	98.57
Putative holin	YP_009278563.1	*Staphylococcus* phage SLPW	98.57
Holin	QWY14507.1	*Staphylococcus* phage JPL-50	98.57
Putative holin	YP_009797781.1	*Staphylococcus* phage vB_SauP_phiAGO1.3	97.86
Holin	YP_004957430.1	*Staphylococcus* phage BP39	97.14
Putative phage holin	YP_009792346.1	*Staphylococcus* phage PSa3	96.43
Holin	YP_010114661.1	*Staphylococcus* phage LSA2366	94.29
Holin	QCW21995.1	*Staphylococcus* phage SA46-CTH2	92.14
Holin	YP_001491536.1	*Staphylococcus* phage SAP-2	92.14
Holin	NP_817307.1	*Staphylococcus* virus 44AHJD	92.14
Holin	YP_010102819.1	*Staphylococcus* phage SA46-CL1	90.71

### Protein expression of Hol-4086

**Figure 4:**
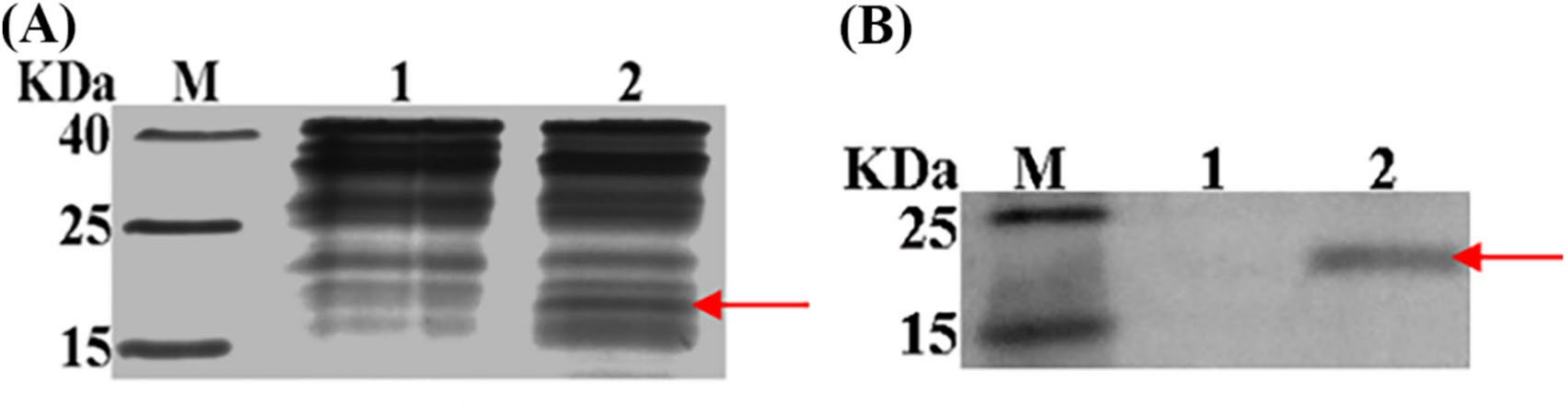
Expression of Hol-4086. **(A)** Sodium dodecyl sulfate-polyacrylamide gel electrophoresis (SDS-PAGE) was performed to detect Hol-4086 expression. **(B)** Western blot analysis of Hol-4086 expression. The lanes are as follows: M, molecular mass marker; 1, *E. coli* BL21-CodonPlus cells harboring pET-28a(+) cultures were collected after induction as a negative control; 2, supernatant from induced cell lysis of recombinants. The red arrow indicates the expressed Hol-4086.

Sequences encoding Hol-4086 were successfully cloned into plasmid pET28a. Hol-4086 was then expressed in *E. coli* cells after induction by IPTG at 37°C. SDS-PAGE and western blotting revealed that the protein had the correct mass and a near homogeneity of 21 kDa, corresponding to the predicted size of the phage Hol-4086 protein (Figure 4).

### Spot test

**Figure 5:**
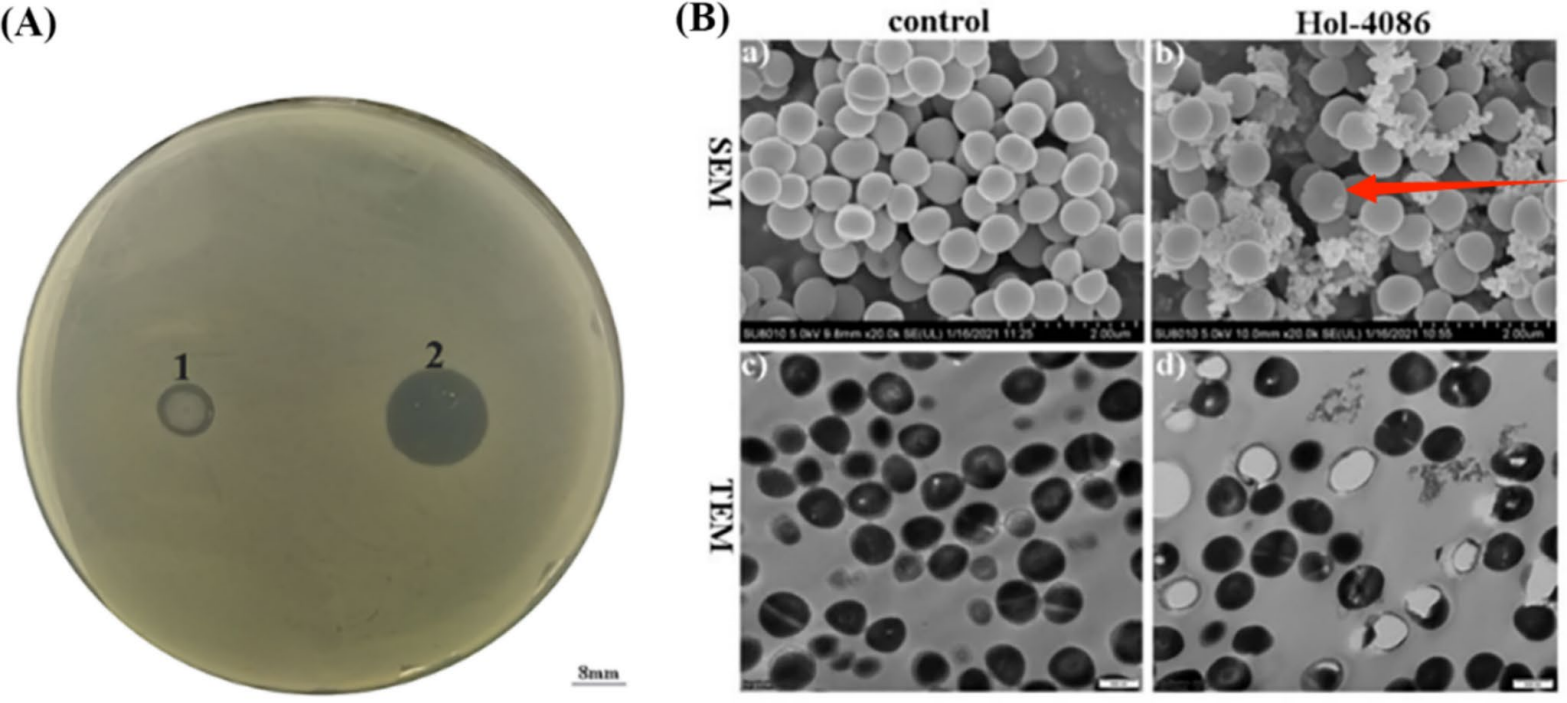
Antibacterial activity of Hol-4086. **(A)** Effects of Hol-4086 on *S. aureus* as observed by spot testing. The holes are as follows: 1, 100 μL uninduced *E. coli* lysates; 2, 100 μL Hol-4086 lysate. **(B)** The effect of Hol-4086 on *S. aureus* observed by transmission electron microscopy (TEM) and scanning electron microscopy (SEM). **(a)** *S. aureus* cells without treatment with Hol-4086 observed by SEM. **(b)** Effects of Hol-4086 on *S. aureus* as observed by SEM. **(c)** *S. aureus* cells without treatment with Hol-4086 observed by TEM. **(d)** Effects of Hol-4086 on *S. aureus* as observed by TEM. Bars: SEM, 2.00 μm; TEM, 500 nm.

After incubation for 12 h at 37°C, a bacteriostatic circle formed on the agar plate that had been treated with expressed Hol-4086 protein, which could not be observed in the negative controls (Figure 5A). These results demonstrate that the expressed Hol-4086 protein has substantial bacteriostatic effects on host bacteria.

### The effect of Hol-4086 on *S. aureus* observed by TEM and SEM

*Staphylococcus aureus* cells were exposed to Hol-4086 and observed using TEM and SEM. TEM and SEM images of the bacterial cells treated with Hol-4086 showed ultrastructural and morphological changes (Figure 5B). According to the SEM observations, Hol-4086 caused the surface shrinkage of *S. aureus*. In addition, TEM revealed that the cell wall of *S. aureus* was destroyed, resulting in the release of cellular contents.

### Biofilm reduction activity of Hol-4086

**Figure 6:**
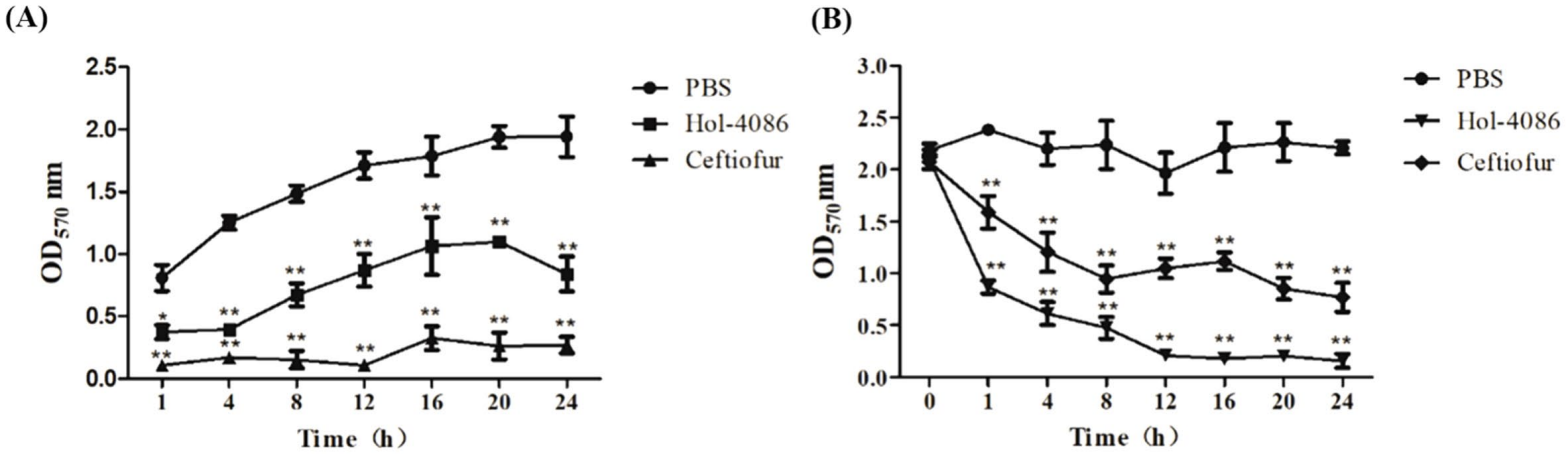
The effects of Hol-4086 on the removal of *S. aureus* biofilms. Biofilm prevention **(A)** and biofilm removal **(B)** by Hol-4086 against *S. aureus* in microtiter plates assays. The biomass amounts are presented as the OD_570_ values. *p < 0.05, **p < 0.01.

Biofilm matrix disruption by Hol-4086 was measured by plate reader at OD_570_. The results of the tests performed for biofilm prevention and removal are shown in Figure 6. As shown in Figure 6A, after incubation with Hol-4086 or antibiotics, biofilm formation was significantly lower than that in the PBS group, and the amount of biofilm after incubation with Hol-4086 was slightly lower than that in the antibiotic group (*p* < 0.01). Hol-4086 also effectively removed *S. aureus* biofilms after incubation for 12 h (*p* < 0.01; Figure 6B).

### Endotoxin test results of Hol-4086

The endotoxin test results showed that the mice in the experimental group and the control group grew well without abnormalities, and the survival rate statistics were shown in Table 2, indicating that there was no toxic effect of Hol-4086 on mice.

**Table 2:** Statistical results of survival rate of mice.

Group	Number of inoculated mice	Number of surviving mice	Survival rate
Holin	5	5	5/5
Control	5	5	5/5

### Colony-forming units

**Figure 7:**
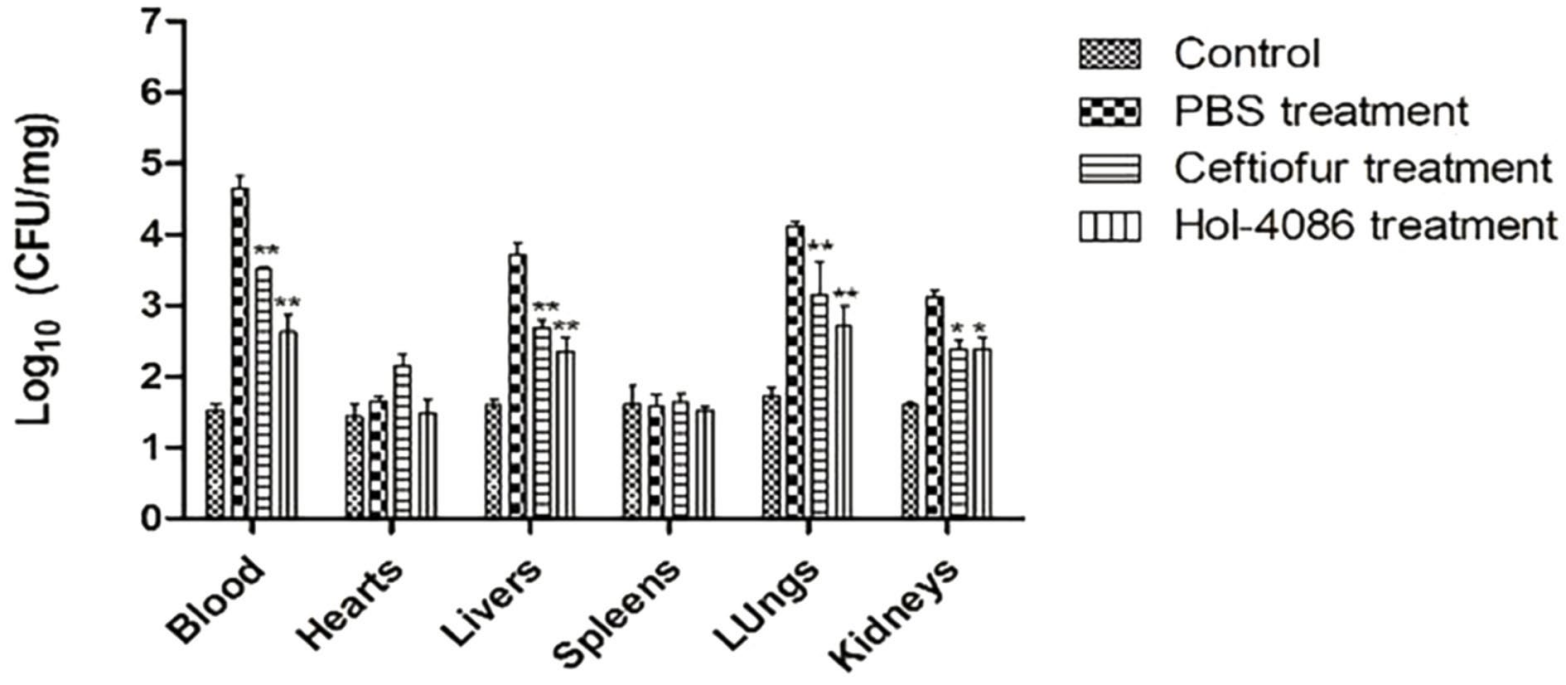
Bacterial loads in the blood and organs of mice. The bacterial loads in the blood, hearts, livers, spleens, lungs, and kidneys of Hol-4086-treated mice decreased by 2.02, 0.16, 1.37, 0.05, 1.39, and 0.73 log units, respectively, 48 h after treatment, compared with PBS group. Error bars represent the mean ± standard error of the mean from three independent replicates, *p < 0.05, **p < 0.01; two-way analysis of variance.

Mice were infected with *S. aureus* to detect the effects of Hol-4086. The healthy mice that were not injected with bacteria or treated were as the control. *S. aureus* counts were determined using the plate-counting method. As shown in Figure 7, the bacterial loads in the blood, heart, liver, spleen, lung, and kidney of Hol-4086-treated mice decreased by 2.02, 0.16, 1.37, 0.05, 1.39, and 0.73 log units, respectively, 48 h after treatment, compared with the PBS group (*p* < 0.01).

### Cytokine levels assay

**Figure 8:**
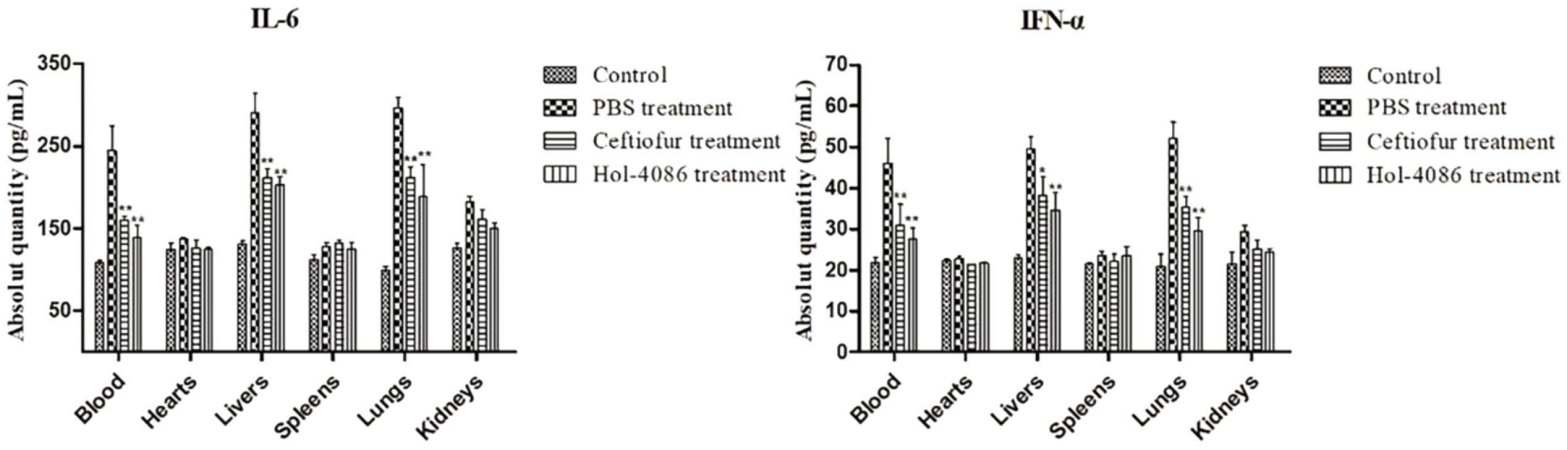
Levels of interleukin-6 (IL-6) and Interferon alpha (IFN-α) from the septicemia model mice after Hol-4086 treatment. Compared with PBS treatment, the ceftiofur and Hol-4086 groups both showed significant decrease in the levels of IFN-α and IL-6. Further, Hol-4086 treatment resulted in slightly lower levels than the antibiotic treatment. Error bars represent the mean ± standard error of the mean from three independent replicates. Compared with PBS treatment, *p < 0.05, **p < 0.01, two-way analysis of variance.

To assay the effect of Hol-4086 on *S. aureus* infection, the levels of IFN-ɑ and IL-6 were determined using ELISA kits. Compared with PBS group, the ceftiofur sodium and Hol-4086 groups both showed significant decreases in the levels of IFN-ɑ and IL-6. The concentration in the Hol-4086 group was slightly lower than that in the antibiotic-treated group (Figure 8). These results demonstrated that Hol-4086 effectively alleviated inflammatory responses to *S. aureus* infection.

### Histopathological analysis

**Figure 9:**
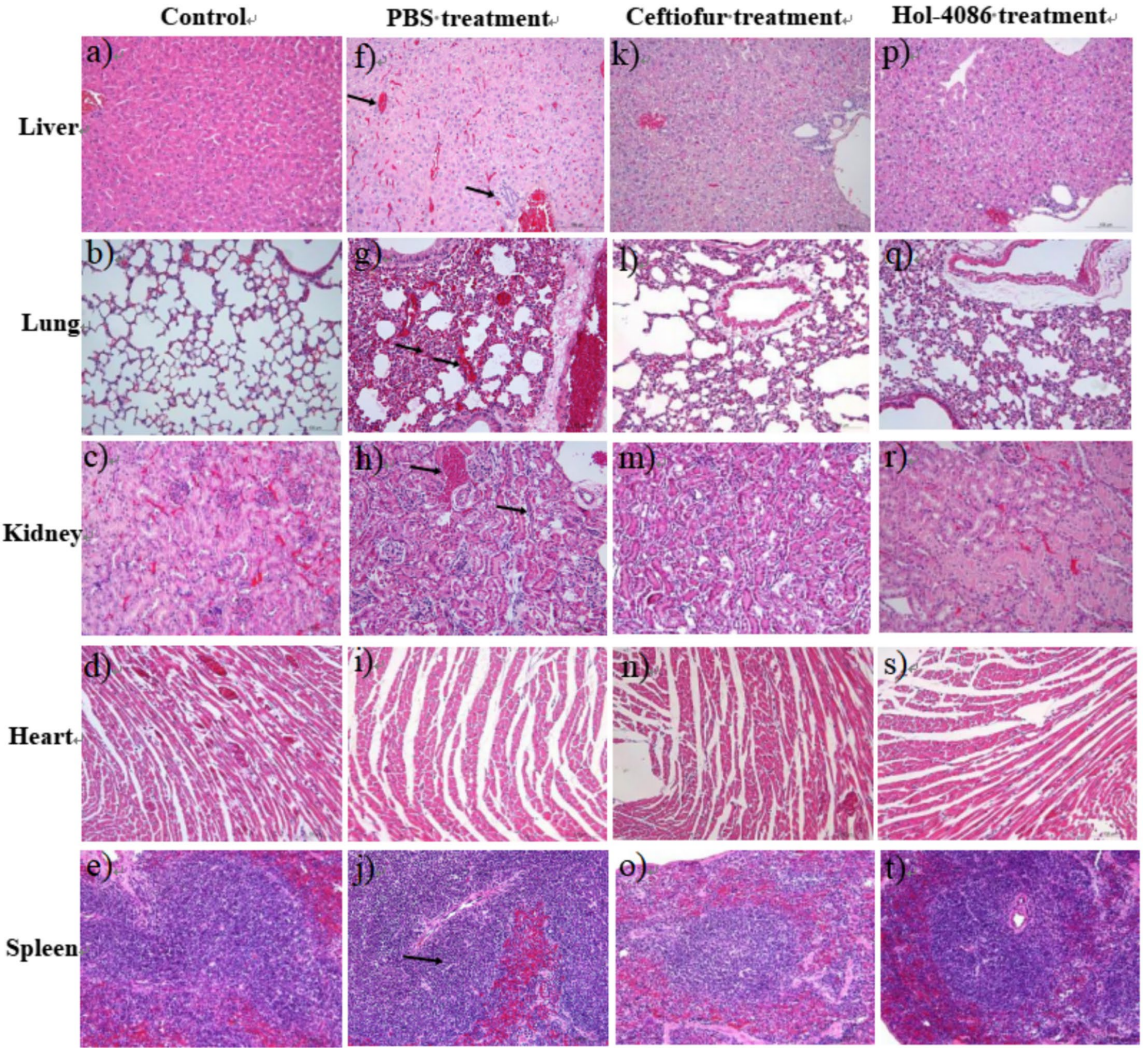
Pathological changes and organ histopathology. Tissue sections were stained with hematoxylin and eosin and observed under a light microscope. Bars, 100 μm. Based on histopathological observations, the liver, lungs, and kidneys of the infected mice treated with phosphate buffered saline (PBS) showed different degrees of pathological changes. After treatment with Hol-4086, the pathological changes were alleviated. **(A–E)** The liver, lungs, kidneys, heart, and spleen of the mice without infection and treatment. **(F–J)** The liver, lungs, kidneys, heart, and spleen of infected mice after treatment with PBS for 48 h. **(K–O)** The liver, lungs, kidneys, heart, and spleen of infected mice after treatment with ceftiofur for 48 h. **(P–T)** The liver, lungs, kidneys, heart, and spleen of infected mice after treatment with Hol-4086 for 48 h.

In the PBS group, congestion, extensive degeneration of hepatocytes, and inflammatory cell infiltration around small bile ducts presented in the livers. The lungs were congested, with alveolar epithelial cell hyperplasia, inflammatory cell infiltration, and local widening of alveolar septa. The kidneys were congested, with extensive degeneration and a small amount of necrosis of renal tubular epithelial cells. There was mild hyperplasia of the white pulp of the spleen (Figures 9F–J). After Hol-4086 treatment, pathological changes in the liver, lungs, and kidneys were alleviated (Figures 9P–T). The hearts of mice in the different groups did not show inflammation or other pathological changes (Figure 9).

## Discussion

The emergence and proliferation of antibiotic-resistant strains of *S. aureus* have stimulated the search for alternative strategies to manage bacterial infections (Pires et al., 2020; João et al., 2021). With rapid developments in molecular biology, several new antibacterial strategies have been proposed. Among these antimicrobial strategies, phage lysis-related proteins are considered the most promising biocontrol agents owing to their specific advantages, such as biosafety *in vivo* (Luo et al., 2020; Srinivasan et al., 2020).

In the present study, we sequenced the whole genome of a *S. aureus* phage with high lytic activity. Analysis of the phage 4086-1 genome indicated that it is a double-stranded DNA consisting of 17,960 bp, which showed high genomic similarity to *S. aureus* phage SLPW (Wang et al., 2016). Phage 4086-1 is structurally similar to the short-tailed phage CAS13, with an icosahedral head and a short, non-constricting tail (Tang, 2019; Cha et al., 2019). ORF12 was predicted to encode a protein composed of 140 amino acids that functioned as a holin (Hol-4086). After prokaryotic expression, the Hol-4086 lyase was shown to have the function of lysing *S. aureus*. In this study, purified protein was not obtained because of the low expression of Hol-4086. The toxic effect of holin on expressing cells may lead to a low exogenous expression output, which is an important reason for restricting the application of holin (Lu et al., 2020). Therefore, it is necessary to find ways to increase the expression output.

Phage lysis-related proteins are potential agents that inhibit bacterial growth (Zhang et al., 2019; Criel et al., 2021; Abdelkader et al., 2019). Endolysins can destabilize the biofilm structure by taking advantage of channels to reach the bacterial cells located inside the biofilm, thereby disrupting the biofilm (Sharma et al., 2018). LysGH15 efficiently prevented biofilm formation and showed notable disruptive activity against 24-h and 72-h biofilms formed by *S. aureus* and coagulase-negative species (Zhang et al., 2018). Many previous studies have shown that holin proteins can induce cracking in host bacteria (Song et al., 2016). The holin protein HolSSE1 and lyase LysSSE1 of *Streptococcus dysenteriae* phage have the efficacy of successfully removing bacterial biofilm. And they have a synergistic antibacterial effect when used together (Lu et al., 2021). In addition, it have shown that the phage holin protein pEF191 shows more efficient biofilm clearance of *Enterococcus faecalis* than the parent phage PEf771, which shows great potential in clinical bactericide (Xiang et al., 2024). In this study, Hol-4086 was characterized and demonstrated bacteriolytic activity against *S. aureus*. We determined the inhibition and removal effect of Hol-4086 on *S. aureus* biofilm. Hol-4086 had a better potential to prevent biofilm formation, most likely due to its rapid bacteriostatic activity prior to biofilm formation, which is similar to holin protein HolSSE1 (Lu et al., 2021). In addition, Hol-4086 was effective in removing *S. aureus* biofilms and could still be effective after 12 h of incubation. Taken together, all these results indicate that Hol-4086 has antibacterial activity against *S. aureus* and can either inhibit biofilm formation or effectively remove biofilms. It was previously reported that Lysin CF-301 was highly efficient for biofilm removal with a 90% minimum biofilm-eradicating concentration (MBEC90) value of 0.25 μg/mL (Schuch et al., 2017). Since we did not obtain the purified protein of Hol-4086 in this study, the dosage of Hol-4086 could not be determined. In the following study, we need to focus on the purification of Hol-4086 to determine its efficiency in removing biofilms and bacteriostatic efficiency.

Phage lysis-related proteins have great potential in clinical treatment. Singh et al. established bacteremia infection in mice by intraperitoneal injection of 10^9^ CFU of *S. aureus*, followed by intraperitoneal or intravenous injection of phage lysis protein; the survival rate of mice was significantly increased, indicating that lysis protein can effectively control the infection caused by bacteremia (Singh et al., 2014). After treatment with the endolysin trxSA1 of phage IME-SA1, bacterial counts in cows were reduced to undetectable levels after 3 days, indicating that trxSA1 could effectively control mild clinical mastitis caused by *S. aureus* (Fan et al., 2016). Some studies have demonstrated that after infection with *S. aureus*, intraperitoneal injection of LysGH15 (50 μg) can provide 100% protection to mice and can significantly reduce the amount of bacteria in the blood and relieve inflammation (Xia et al., 2016; Zhang et al., 2016). In this study, we assayed the function of Hol-4086 and intraperitoneally injected mice with *S. aureus* to establish sepsis infection. We found that Hol-4086 significantly reduced bacterial loads in the blood, liver, and lungs, effectively reduced pathological damage, and alleviated inflammatory responses. These findings suggest that Hol-4086 is an active treatment *in vivo* and may be a potential therapeutic tool.

As a new antibacterial agent, Hol-4086 has similar functional activity and therapeutic effect to phage (Sharma et al., 2018; Zhang et al., 2018; Singh et al., 2014), which shows a great potential on *S. aureus* infection. Hol-4086 was still effective in removing *S. aureus* biofilms after 12 h of incubation, which is expected to be used as an environmental antimicrobial in the future. Endotoxin test showed that Hol-4086 did not cause damage to mice, which can provide a new idea and technical means for the treatment of clinical drug-resistant bacteria. More experiments still need to be carried out to explore the effect of Hol-4086 on eukaryotic cells before clinical practice. In addition, Hol-4086 expressed in *E. coli* has an inhibitory effect on the host bacteria itself, resulting in a low expression level and no purified protein can be obtained. Therefore, the influence of expression system and elements on the expression level should be further optimized in subsequent experiments for clinical treatment.

## Conclusion

In summary, as a new type of antibacterial agent, Hol-4086 has a functional activity similar to that of phages, with no bacterial resistance or side effects. Hol-4086 has good therapeutic effects against *S. aureus* infections and can alleviate inflammation induced by infection. Thus, Hol-4086 may serve as a new therapeutic option for drug-resistant *S. aureus* in clinical settings.

## Data Availability

The datasets presented in this study can be found in online repositories. The names of the repository/repositories and accession number(s) can be found: https://www.ncbi.nlm.nih.gov/genbank/, PP541615; https://www.ncbi.nlm.nih.gov/genbank/, PP477054.

## Footnotes

^1^ http://www.cbs.dtu.dk/services/TMHMM/

^2^ http://blast.ncbi.nlm.nih.gov/Blast.cgi

^3^ https://www.ncbi.nlm.nih.gov/Structure/cdd/wrpsb.cgi
